# Supplementary figures and images for: A feasibility study on implementing pre-emptive pharmacogenomics testing in outpatient clinics in Singapore (IMPT study)
Source: Pharmacogenomics J. 2025 Mar 12;25(1-2):7. doi: 10.1038/s41397-025-00366-1 (PMC11903297; doi:10.1038/s41397-025-00366-1)

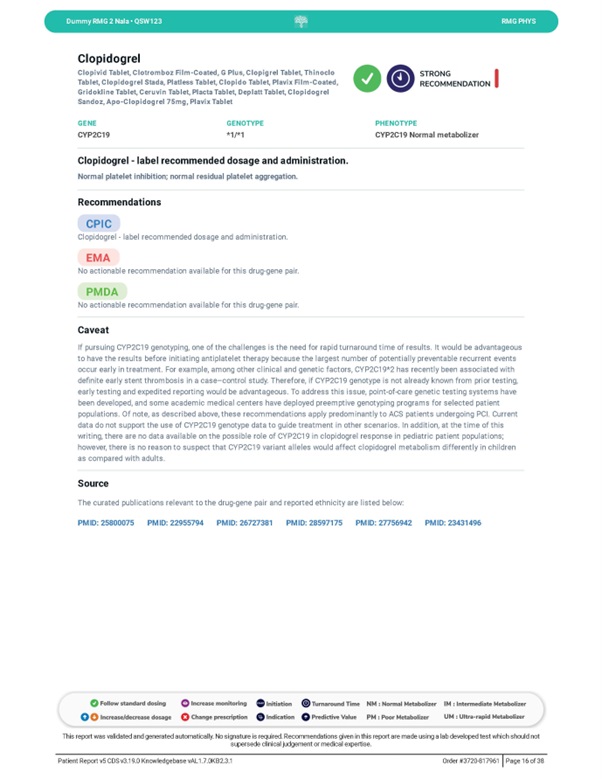

Supplement: Supplementary file 2 — Supplementary Material S1: Sample pharmacogenomics report [file 41397_2025_366_MOESM2_ESM.jpg]
